# Recruitment Kinetics of Tropomyosin Tpm3.1 to Actin Filament Bundles in the Cytoskeleton Is Independent of Actin Filament Kinetics

**DOI:** 10.1371/journal.pone.0168203

**Published:** 2016-12-15

**Authors:** Mark A. Appaduray, Andrius Masedunskas, Nicole S. Bryce, Christine A. Lucas, Sean C. Warren, Paul Timpson, Jeffrey H. Stear, Peter W. Gunning, Edna C. Hardeman

**Affiliations:** 1 Cellular and Genetic Medicine Unit, School of Medical Sciences, UNSW Australia, Sydney, New South Wales, Australia; 2 The Kinghorn Cancer Center, Garvan Institute of Medical Research, Sydney, New South Wales, Australia; University of Bonn, GERMANY

## Abstract

The actin cytoskeleton is a dynamic network of filaments that is involved in virtually every cellular process. Most actin filaments in metazoa exist as a co-polymer of actin and tropomyosin (Tpm) and the function of an actin filament is primarily defined by the specific Tpm isoform associated with it. However, there is little information on the interdependence of these co-polymers during filament assembly and disassembly. We addressed this by investigating the recovery kinetics of fluorescently tagged isoform Tpm3.1 into actin filament bundles using FRAP analysis in cell culture and *in vivo* in rats using intracellular intravital microscopy, in the presence or absence of the actin-targeting drug jasplakinolide. The mobile fraction of Tpm3.1 is between 50% and 70% depending on whether the tag is at the C- or N-terminus and whether the analysis is *in vivo* or in cultured cells. We find that the continuous dynamic exchange of Tpm3.1 is not significantly impacted by jasplakinolide, unlike tagged actin. We conclude that tagged Tpm3.1 may be able to undergo exchange in actin filament bundles largely independent of the assembly and turnover of actin.

## Introduction

The actin cytoskeleton is a dynamic meshwork of filaments that are involved in most biological processes. Precise regulation of actin filaments is crucial to maintain the integrity of these processes and involves interactions with various actin-associated and -binding proteins including the tropomyosins (Tpms) [1]. Indeed, most non-muscle or cytoskeletal actin filaments are comprised of polymers of actin and Tpm. Tpms are α-helical coiled coil dimers that form two continuous parallel polymers along the major grooves of the actin polymer and ‘bind’ to actin via ionic interactions [2]. Over 40 cytoskeletal Tpm isoforms exist in mammalian cells and define the functional capacity of actin filaments by regulating the interaction of actin filaments with actin-binding proteins and myosin motors [1, 3, 4]. Studies have indicated that the formins specify, at least in part, which Tpm isoform is incorporated into an actin filament [5, 6] which suggests that Tpm polymer formation is dependent on actin dynamics.

Distinct actin filament populations have been identified in cultured cells. There are at least four different categories of stress fibers: dorsal, ventral, transverse arcs and the perinuclear cap [7]. In addition, two actin sub-populations have been identified at the cell cortex distinguished by very different turnover and polymerization rates [8]. Analysis of isoform-specific Tpm dynamics associated with stress fibers has been carried out using fluorescence recovery after photobleaching (FRAP) and revealed different recovery rates. When YFP/GFP-tagged Tpms 1.7, 3.1 and 1.9 (previously Tm3, Tm5NM1 and Tm5b, respectively; [9]) were compared, Tpm3.1 had a higher rate of recovery on actin stress fibers [10]. Of the four Tpm isoforms shown to be essential for stress fiber formation, 1.6 (previously Tm2), 1.7, 3.1 and 4.2 (previously Tm4), Tpm4.2 had a faster recovery than the other 3 isoforms [6]. There is no information about the relationship between actin and Tpm dynamics in filaments of the cytoskeleton; however, recent data on the assembly of pre-myofibrils suggests that there is not an absolute relationship between actin and Tpm turnover [11]. A secondary question is whether the placement of a tag impacts Tpm dynamics since no comparison has been carried out between N and C-terminal tagged Tpm constructs.

In this study we used FRAP to investigate the interrelationship of Tpm and actin dynamics *in vitro* in cultured cells and *in vivo* in tissues. In particular, we focused on isoform Tpm3.1 that is known to stabilize actin filaments by reducing depolymerisation [12] as well as recruiting myosin motors [13]. We used fluorescent protein-tagged Tpm3.1 and actin and examined Tpm3.1 vs actin recovery in dorsal/ventral stress fibers in mouse embryo fibroblasts (MEFs) and in apical/cortical fibers in rat salivary gland acinar cells. Recovery of Tpm3.1 was determined on actin filaments perturbed with the actin-targeting drug jasplakinolide, which promotes actin filament nucleation and stabilization [14, 15]. We also investigated the impact of placing a fluorescent tag at either the N- or C-terminus of Tpm3.1 on the fidelity of its localization and recovery kinetics. Our data is compatible with a continuous dynamic exchange of Tpms occurring on actin filaments that is independent of actin filament dynamics and the location of the tag on Tpm3.1.

## Materials and Methods

### Cell culture, transfection and drug treatment

MEFs were isolated from day 13.5 C57Bl/6 mouse embryos and cultured as previously described [16]. Cultured cells were maintained in Dulbecco’s modified Eagle medium (DMEM) with 10% (v/v) foetal bovine serum (FBS) at 37°C, 5% CO_2_. For imaging experiments, MEFs at passages 1–3 were seeded into Fluorodish^™^ tissue culture dishes (World Precision Instruments Pty Ltd) and grown to 70–90% confluency. Cell transfections were performed using Lipofectamine 3000 reagent (Life Technologies) and plasmid DNA according to the manufacturer’s instructions. Jasplakinolide (Sapphire Bioscience Pty Ltd) was added at a final concentration of 7 μM from a 1mM stock prepared in DMSO. FRAP analysis was performed within 10 s after addition of the drug.

### Tpm3.1 and actin constructs

The sequence encoding mNeonGreen (a gift from Jiwu Wang, [17]) was inserted at either the N- or C-terminus of human Tpm3.1, separated by a 10 amino acid linker motif (GGGGSGGGGS), and cloned into pcDNA3.1 under control of a CMV promoter (GeneArt, Invitrogen). pCAG-GFP-actin was a gift from Ryohei Yasuda (Addgene plasmid #21948) [18]. Lifeact-RFP was a gift from Roland Wedlich-Soldner [19].

### Immunohistochemistry

Transfected wildtype B6 and B6-*Tpm3*^tm2(Δ9d)Pgun^ MEFs [20] (mice lack exon 9d of the *Tpm3* gene resulting in the knockout of isoforms Tpm3.1 and Tpm3.2) were fixed in 4% PFA at RT for 30 min, permeabilized in ice cold methanol for 30 min, blocked in 2% BSA in PBS at RT for 60 min. The cells were incubated with CG3 in 2% BSA (mouse monoclonal, 1:25 [21]), which recognizes the 1b exon from the TPM3 gene (1 h, RT) followed by Alexa-647 conjugated donkey anti-mouse secondary antibodies (1:400 in PBS). Cells were washed 3 times with PBS and imaged using a Zeiss 880 confocal using a 63x/1.4 NA objective and sequential excitation with 488 nm and 633 nm lasers. The raw image data was deconvolved using the Airyscan processing algorithm that is included with the Zeiss Zen software package.

## Western Blotting

Protein was extracted from transfected and non-transfected MEFS in RIPA buffer and analysed by SDS-PAGE and western blotting as described previously [22]. Protein concentration was estimated using Precision Red (Cytoskeleton, Inc). Equal amounts of protein (30 μg) were resolved on a 12% SDS-PAGE gel before electro-transfer to PVDF membranes. Non-specific binding on the blot was blocked with blocking buffer, 1% BSA in TBST (100 mM Tris-Cl, pH 7.5. 150 mM NaCl with 0.05% Tween 20). Tpm 3.1 was recognised using monoclonal ab CG3 (1:200 in blocking buffer) [22] and secondary antibody rabbit anti mouse Ig-conjugated horseradish peroxidase (Abcam) (1:10,000 in blocking buffer). Primary antibody was incubated overnight and secondary for 2 h with 4 x 15 min washes. Blots were developed with the Western lightening Chemiluminescence reagent (Perkin Elmer Life Sciences; Boston, MA) and exposed to x-ray film. Equal protein loading was examined by staining the protein gel blots with 0.1% (w/v) Coomassie blue R350, 20% (v/v) methanol and 10% (v/v acetic acid).

### Live cell imaging and FRAP assay

Live cell imaging was performed on a Nikon A1 inverted scanning confocal microscope fitted with a Nikon Plan Apochromat λ 60x oil immersion objective with an NA of 1.4 and an Okolab incubation chamber equilibrated to 37°C. The mNeonGreen (λem 516 nm) constructs were excited with a 488-nm laser. Transiently transfected cells with a low level of tagged Tpm3.1 expression were chosen for FRAP analysis. For time-lapse imaging, frames were acquired at 516 ms/frame at 256 pixel resolution, 300 nm per pixel, and imaged at 1 Hz. FRAP zones were bleached with a single 120 ms pulse using a 488-nm laser. 3–5 reference frames were acquired per cell, followed by a single bleach pulse, followed by image acquisition at 1 Hz for 120 s.

### Animal transfections

Animal experiments were performed in accordance with the NSW Animal Research Act (1985) and Australian National Health and Medical Research Council (NHMRC) ‘Code’ 8^th^ edition (2013). All experiments were approved by the UNSW Australia Animal Care and Ethics Committee under applications 12-120B, 15-103A and 14-92A. Male Wistar rats weighing 150–225 g were obtained from the Animal Resources Centre, Perth, Australia and allowed to acclimatize for 1 week. Rats were anesthetized and salivary glands (SGs) transfected as previously described [23] with the following modifications: 24 μg of plasmid DNA was mixed with *in vivo*-jetPEI^®^ (Polyplus Transfection) with 100 μL of 10% w/v glucose according to the manufacturer’s instructions.

### Intravital imaging and FRAP assay

Rats were anesthetized and salivary glands externalized and prepared for intravital imaging as previously described [23]. Intravital imaging was performed on a Nikon A1 inverted laser scanning confocal microscope fitted with a Plan Apochromat WI DIC N2 60x water objective with an NA of 1.27, an Okolab incubation chamber and a custom made stage insert. mNeonGreen constructs were excited with a 488 nm laser. For time-lapse imaging, frames were acquired at 477 ms/frame at 256 pixel resolution, 100 nm per pixel, and imaged at 1 Hz. FRAP zones were bleached with a single 120 ms pulse using a 488 nm laser. 3–5 reference frames were imaged per cell, followed by a single bleach pulse at a defined ROI, followed by acquisition at 1 Hz for 120 s.

### Tissue sectioning and immunostaining

Salivary glands were dissected from adult male C57Bl/6 mice, immersed in Tissue-Tek and frozen in isopentane cooled in liquid nitrogen. 10 μm sections were cut using a Cryostat (Leica CM1950) at -20°C, fixed for 30 min in 1% PFA in PBS at 4°C, treated for 5 min with MeOH at -20°C, and blocked with 5% goat serum, 5% FBS, 1% BSA in PBS for 2 h, RT. Mouse mAb 2G10 against Tpm3.1/3.2 (1:200 in blocking buffer) [22] was added for 12 h, RT, and then secondary antibody conjugated with AlexaFluor488 (mouse; Invitrogen) (1:750 in blocking buffer) for 1 h, RT. Images were taken with an Olympus FV1000 confocal microscope. Transfected salivary glands were excised, immersed in 50 μg/mL 10kDa dextran-Alexa647 conjugate (Invitrogen) for 10 min, placed on a glass coverslip and the surface imaged immediately as for intravital imaging.

### Image and data processing

Images were taken using NIS Elements software. Image processing and data extraction was performed using ImageJ (v1.47n). Data processing and analyses were performed using Microsoft Office Excel. All statistical *p* values were obtained from unpaired t-tests using Graphpad Prism 6. FRAP curves were normalized to the minimum and maximum fluorescence values using a value range of 0 (minimum fluorescence) to 1 (maximum fluorescence). Data from normalized FRAP curves were then fitted with a double exponential equation using the IgorPRO6 software complemented with the K_FRAPcalc version 9 procedure (Kota Miura, EMBL-Heidelberg, Germany) to derive half-times and mobile fractions. Fits obtained using either single or double exponential FRAP recovery model were compared using the extra sum-of-squares F test in Graphpad Prism 6 to determine whether the double exponential recovery provided a statistically significant improvement in the quality of the fit, accounting for the extra degrees of freedom in the more complex model. 95% confidence intervals on the fitted parameters were estimated using Graphpad Prism 6 using the delta method.

Radially averaged spatial recovery profiles were obtained using a custom MATLAB (Mathworks) script by computing the average intensity at increasing distances from the centre of the bleached region and normalising to the pre-bleach intensity. The spatial profile of the recovery was fitted to a Gaussian model as previously described to determine the width of the bleached region [24, 25].

## Results and Discussion

### Visual characterization of N- and C-terminal tagged Tpm3.1 constructs

**Fig 1:**
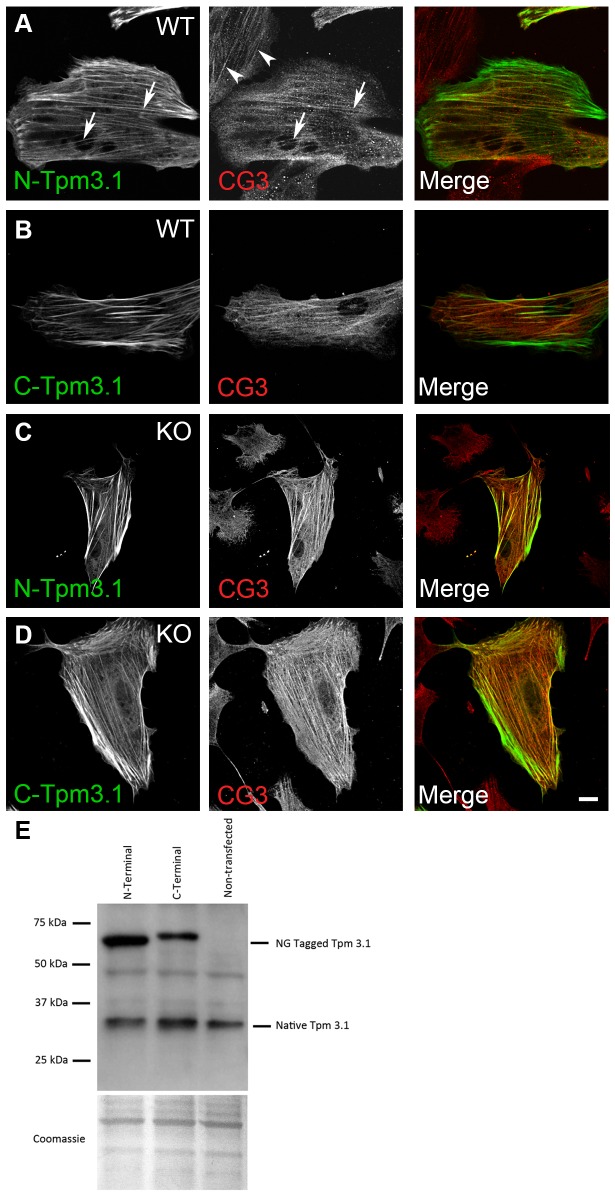
N- and C-terminal tagged Tpm3.1 both localize to stress fibers in mouse embryo fibroblasts. Tagged Tpm3.1 constructs were transfected into wild type and Tpm3.1/3.2 knockout mouse embryo fibroblasts (MEFs) and visualized by confocal microscopy (A and C) N-terminal tagged Tpm3.1 and (B and D) C-terminal Tpm3.1. Tpm3.1 was visualized using the CG3 antibody that recognises all isoforms from the TPM3 gene. (E) Western blot showing expression of the tagged Tpm3.1 constructs and endogenous Tpm 3.1 in primary wild type MEFs as detected by the CG3 antibody. Scale bar = 10 μm.

N-terminal (N-Tpm3.1)- and C-terminal (C-Tpm3.1)-tagged Tpm3.1 were transfected into wild type and Tpm3.1/3.2 knockout MEFs and the tagged constructs localized predominantly to stress fibers in both cell types (Fig 1A–1D). These cells were co-stained with the CG3 antibody that detects all isoforms expressed from the TPM3 gene as well as both N-Tpm3.1 and C-Tpm3.1 (Fig 1A–1E). The CG3 antibody detects stress fibers in both untransfected (arrowheads Fig 1A) and transfected wild type MEFs (arrows Fig 1A). These stress fibers co-localize with the tagged proteins, however there are regions, particularly at the ends of stress fibers and regions of high tag density that do not co-localize with the antibody staining. This is not due to a failure of the antibody to recognise the tagged Tpm3.1 based on both Western blot results (Fig 1E) and detection of the transfected tagged proteins in Tpm3.1/3.2 knock out cells (Fig 1C and 1D). We hypothesize that there may be steric hindrance of the antibody epitope in these regions and as a result of this only the central region of stress fibers was analysed in further experiments.

This result indicates that both types of tagged Tpm3.1 proteins have very similar localization patterns to each other that is surprising because there is extensive evidence that the N-termini of Tpms are crucial for Tpm function. From studies on muscle Tpms, it is known that the N-terminal residues are highly conserved [26, 27] and that acetylation of a methionine is required for normal function [28], regulation of actomyosin ATPase with troponin [29] and Tpm dimer formation [30]. Mammalian cytoskeletal Tpms in contrast do not require acetylation to bind actin [31]. In yeast, tagging the N-terminus of Tpm prevents acetylation of the N-terminal methionine causing the tagged protein to mis-localize [5] and recent studies indicate that care must be taken in interpreting functional outcomes using tagged Tpms [32]. Nevertheless, expression of transfected N-terminally tagged Tpm3.1 shows biological activity in a cell motility assay in MEFs, but proving biological equivalence of tagged and untagged Tpm3.1 has not been established [33]. In contrast, the C-terminus has a more variable amino acid sequence and a more flexible structure [34, 35], thus tagging the C-terminus of Tpm3.1 is expected to cause less perturbation to the normal activity of the protein, however in this experiment we observed localisation of both constructs to stress fibers.

### Tpm3.1 has a rapid rate of exchange on stress fibers

**Fig 2:**
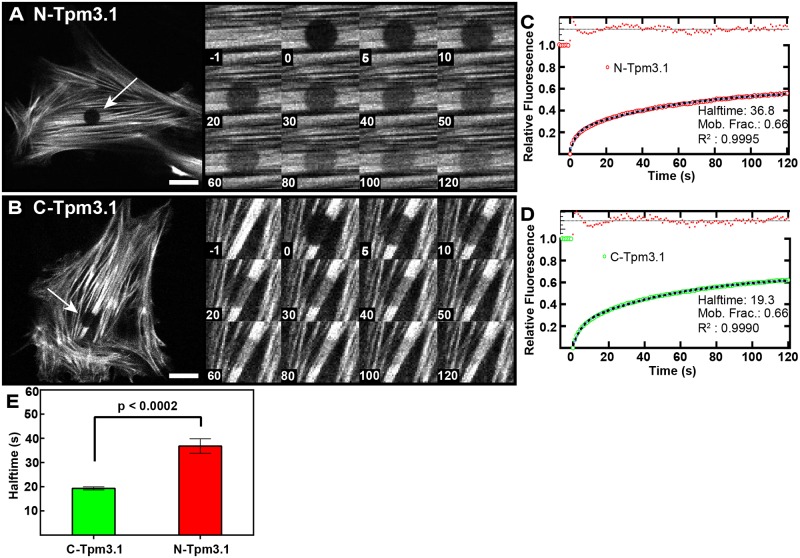
N- and C-terminal tagged Tpm3.1 constructs have similar mobile fractions but dissimilar recovery rates. (A,B) Representative images of FRAP assay in MEFs transfected with either N- or C-Tpm3.1. FRAP zones (white arrows) were bleached and cells imaged at 1 fps for 2 min. (inset A,B). Enlarged images of FRAP zones over time (s). (C,D) FRAP curves of N- or C-Tpm3.1 transfected MEFs. (E) Half-times of N- and C-Tpm3.1 recovery (see also S1 Table). Data obtained from 6 experiments, 3–15 cells per experiment. Error bars are +/- *SEM*. Scale bars = 10 μm.

We elected to use FRAP assay to assess the dynamics of recruitment of Tpm3.1 into actin filament bundles in relation to actin dynamics using constructs tagged at either the N- or C-termini. By photobleaching zones containing stress fibers in the interior of the cell and monitoring the fluorescence recovery we are able to characterise the kinetics of Tpm3.1 on a cell-by-cell basis (Fig 2A and 2B; S1 Table). In a typical FRAP experiment, the fluorescence will recover due to movement of unbleached molecules into the bleached zone and eventually reach a plateau. The level of the plateau provides information about the fraction of molecules that are mobile in the bleached zone (the ‘mobile fraction’) while the shape of the recovery provides information about the number and rates of the dynamic processes leading to recovery, for example diffusion and exchange with a cytoplasmic pool. To determine the number of recovery processes present, we fitted recovery curves from both N- and C-terminal tagged constructs to a single- and double-exponential FRAP recovery model. We found that the double exponential model provided a statistically significant improvement in the quality of the fit compared the single exponential model. The C-terminal fit R^2^ values were calculated at 0.9835 for the single exponential fit compared with 0.999 for the double exponential fit (p<0.0001) with the N-terminal R^2^ values calculated at 0.9860 and 0.9995 for the single and double exponential fits respectively (p<0.0001), suggesting there are two dominant processes contributing to the recovery. We hypothesised that there are three candidate processes potentially contributing to the recovery: (1) diffusion of unbound tropomyosin molecules in the cytoplasm, (2) exchange of tagged- for untagged-Tpm3.1 on actin filaments which are exposed to the cytoplasm and (3) relatively slower exchange of tagged- for untagged-Tpm3.1 on actin filaments located in the interior of stress fiber filament bundles which are not in direct contact with cytoplasm. To determine whether the fast recovery component observed was associated with diffusion or exchange of Tpm3.1 between actin filaments and the cytoplasm we examined in more detail the spatial profile of the recovery process. These processes will produce distinct spatial recovery profiles as illustrated schematically in S1A and S1B Fig. Recovery due to diffusion will show an increase in the width of the bleached region over time as bleached and unbleached molecules diffuse, while the width of the bleached region will remain unchanged in an exchange process [24, 25]. We computed radially averaged spatial recovery profile by averaging over the bleached regions of a number of N- and C-terminal tagged Tpm3.1 transfected cells, shown in S1C and S1D Fig. In both cases the spatial recovery profile is consistent with an exchange-based recovery. In line with this, we calculated the width of the bleached region over time in both cases by fitting to a Gaussian profile and found no increase in the width over time. We therefore concluded that diffusion does not contribute significantly to the observed recovery and the two recovery processes are associated with exchange of Tpm3.1 between cytoplasm exposed and shielded filament bundles, respectively. This conclusion is supported by visual inspection of the movies and micrographs (Fig 2A and 2B); association of tagged-Tpm3.1 with filament bundles starts almost immediately after bleaching. We hypothesise that the relatively slower recovery process is due to packing of filaments in the interior of stress fibers leading to potential steric impediments to complete exchange, independent of filament turnover.

Initial preliminary data using cells transfected with Tpm3.1-YFP were acquired for 5 min, but no stable plateau was observed. This is due to the inevitable movement of the cells during imaging (typically at 1.5 to 2 min), resulting in unbleached structures being imported into the FRAP zone with time. This is illustrated in S1 Movie, showing movement of the stress fibers through the FRAP zone after 2 min as well as the stress fiber moving in and out of the focal plane making analysis after 2 min unreliable. Therefore, to minimise motion artefacts in the FRAP recovery data, all photobleaching experiments were performed for 2 min post-bleach and fitted with a double exponential recovery model to determine the mobile fraction and recovery half-times. Although the recovery is not fully complete at 2 min, we found that the data recorded up to this point was able to sufficiently constrain the model and so obtain an accurate estimate of the mobile fraction; for N- and C- terminal tagged Tpm3.1 the spread of the 95% confidence interval on the estimated mobile fraction ranged from ±1% to ±5%.

Recovery curves for C-Tpm3.1 and N-Tpm3.1 showed identical mobile fractions of 66% in MEFs (Fig 2C and 2D), and no statistical difference between the rate of fast exchange or relative contributions of slow and fast exchange, indicating that the fast exchange process is not affected by the tag location. N-Tpm3.1, however, exhibited a significantly longer slow recovery half-time than C-Tpm3.1 (64.6±14.9s vs 40.2±3.2s, *p* = 0.017), indicating that this slower exchange process is inhibited by the N-terminal tag. The slower recovery half-time observed for N-Tpm3.1 (Fig 2E) is consistent with the finding that muscle Tpm with an 80 residue N-terminal fusion peptide binds with an affinity slightly greater than a non-fusion variant and many-fold greater than unacetylated Tpm [29]. The fusion Tpm failed to be regulated by Troponin T and did not regulate the actomyosin Mg ATPase. Inserting the tag in the N-terminus could potentially alter normal Tpm regulation and binding through steric hindrance thus reducing the rate of N-Tpm3.1 incorporation into actin filaments. For these reasons we selected C-Tpm3.1 as construct of choice for subsequent cellular experiments.

### Intravital imaging and FRAP analysis of Tpm3.1 recruitment in transfected rat salivary glands

**Fig 3:**
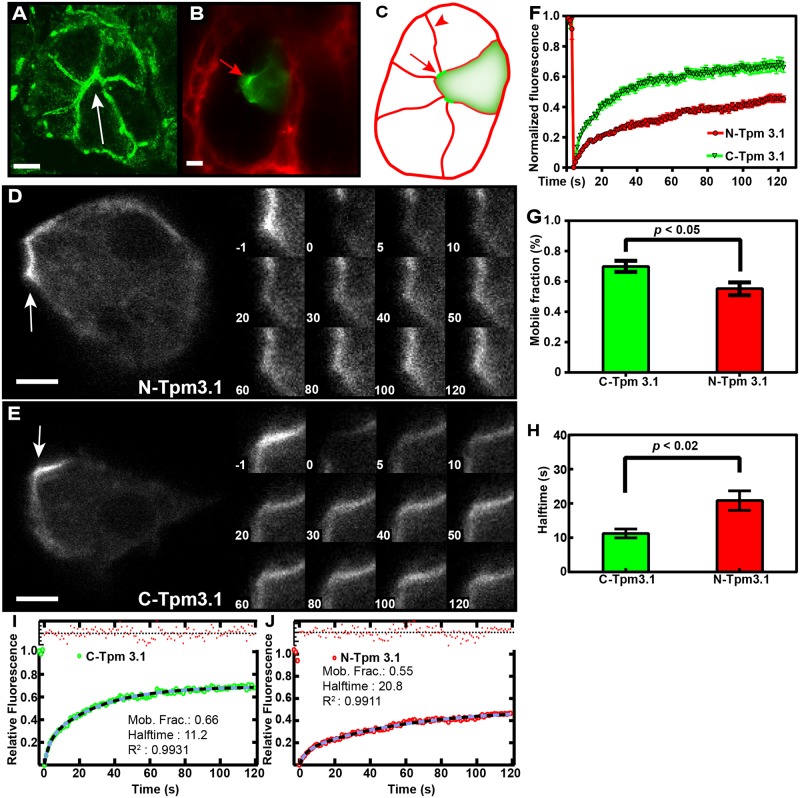
Intracellular intravital imaging of the kinetics of N- and C-terminal tagged Tpm3.1 constructs transfected into rat salivary gland acinar cells. (A) Confocal image of an acinus from rat submandibular salivary gland section stained with anti-Tpm3.1 (2G10) antibody. Tpm3.1 is enriched on the apical plasma membranes that form the canaliculi of acinar cells (white arrow). (B) Confocal image of a C-Tpm3.1 transfected cell (arrow) in a single acinus of a rat salivary gland *in situ*. Extracellular space outside the acinus was stained with 10kDa dextran Alexa 647 conjugate. (C) Illustration of the transfected acinar cell in (B), arrow shows apical membrane/canaliculi, arrowhead shows basolateral membrane. (D,E) Intravital microscopy and FRAP analysis of N- and C-Tpm3.1 constructs in live transfected rats. Numbers indicate time in sec. White arrows indicate FRAP zones on the canaliculi of rat acinar cells. (F) FRAP curves for N- and C-Tpm3.1. (G) Mobile fraction of N- and C-Tpm3.1. (H) Half-times for N- and C-Tpm3.1. (I, J) Curve fits for N- and C-Tpm3.1. 11–16 cells assayed from at least 3 animals per construct (see also S2 Table). Error bars are +/- *SEM*. Scale bars = 5 μm.

To confirm these observations *in vivo*, we applied FRAP analysis to transfected salivary gland acinar cells in live rats using intracellular intravital microscopy. We chose the rat salivary gland as our *in vivo* model because of its tractability for intravital imaging and genetic manipulation [23]. Salivary acinar cells express endogenous Tpm3.1 that is highly enriched at the apical membranes that are arranged into canaliculi (Fig 3A, white arrow). Therefore, intravital FRAP assay was carried out on the apical regions of transfected cells (Fig 3B and 3C). Both N- and C-terminally tagged proteins were localized at the apical membranes of acinar cells (Fig 3D and 3E, white arrows). FRAP recovery kinetics showed similar trends to that seen in cultured MEFs although C-Tpm3.1 has a significantly higher mobile fraction (66%) compared to N-Tpm3.1 (55%) (Fig 3F, 3G, 3I and 3J) (S2 Table). Therefore, the tagged proteins display similar but distinct activities *in vitro* and *in vivo*, in quite dissimilar actin filament structures–stress fibers vs apical filament bundles. As observed with MEFs, the half-life of recovery of C-Tpm3.1 was half of that seen with N-Tpm3.1 (Fig 3H).

**Fig 4:**
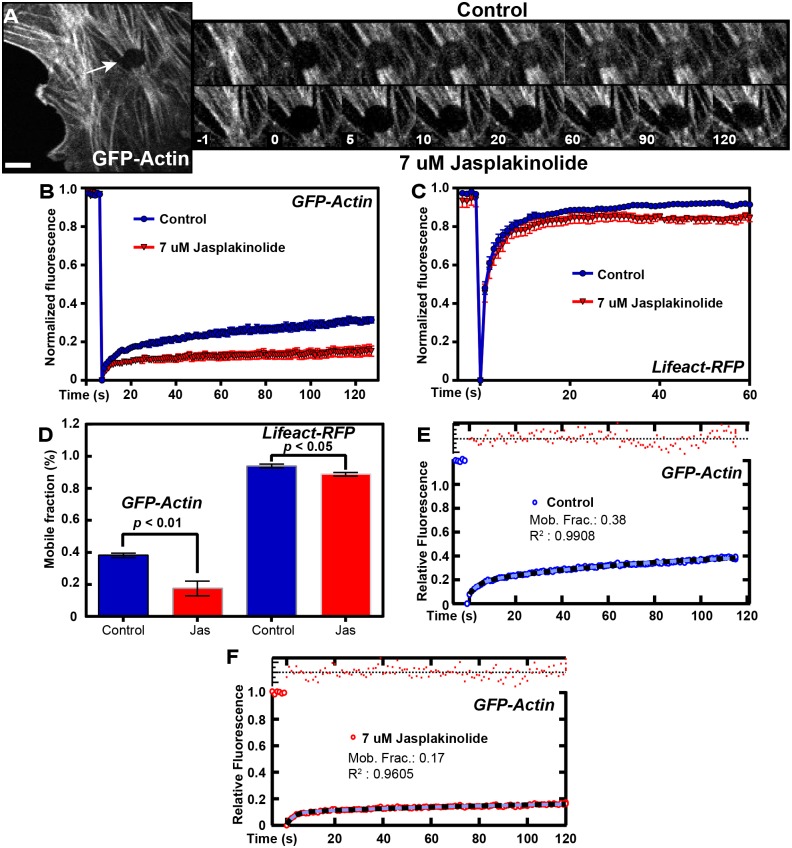
The majority of actin in stress fibers is stable. (A) Representative image and FRAP sequence of MEFs transfected with GFP-beta-actin. FRAP zone indicated by white arrow. Top panel: FRAP sequence of untreated control cells. Bottom panel: FRAP sequence after treatment with 7 μM jasplakinolide. (B) FRAP curves for GFP-actin in control and jasplakinolide treated condition. (C) FRAP curves for Lifeact-RFP in control and jasplakinolide treated condition. (D) Mobile fractions of control and drug treated GFP-actin and Lifeact-RFP (see also S3 and S4 Tables). (E) Curve fits for GFP-Actin control. (F) Curve fit for GFP-Actin treated with jasplakinolide. Data obtained from 3 separate experiments, 2–8 cells per experiment. Error bars are +/- *SEM*. Scale bars = 5 μm.

Comparison of recovery curves for the C-terminal tagged Tpm3.1 with Lifeact in MEFs indicates that Tpm3.1 has a high mobile fraction although not as high as Lifeact (Figs 2D and 4C). Because Lifeact engages in rapid exchange binding to actin filaments the mobile fraction is over 90% and has a very short half-time (S3 Table). However, the fact that Tpm3.1 also has a short half-time and a high mobile fraction both in MEFs and in acinar cells *in vivo* suggests that most of the Tpm3.1 is engaging in rapid exchange with a soluble pool. In contrast, GFP-actin shows a relatively slow recovery curve (Fig 4A and 4E) (S4 Table) that raised the possibility that Tpm3.1 may be exchanging independent of actin filament turnover.

### Impact of the actin-filament stabilising drug jasplakinolide on Tpm3.1 recruitment into stress fibers

Wang et al. [11] recently showed that C-terminal tagged muscle Tpm exchange is much less sensitive to jasplakinolide than tagged actin in pre-myofibrils suggesting that Tpms can exchange independent of actin. Since our results suggest a similar conclusion we tested this in the MEF cytoskeleton by examining the cytoskeletal Tpm3.1. Our approach was to inhibit actin filament kinetics in MEFs using jasplakinolide to stabilise the actin filaments and measuring Tpm3.1 kinetics in stress fibers using FRAP analysis. Using this strategy, we specifically manipulated the kinetics of actin filaments; therefore, any recovery following photobleaching should reflect Tpm3.1 dynamics independent of actin dynamics. First, we established the conditions under which actin filaments were disrupted with drug treatment. We found that FRAP analysis of MEFs transfected with GFP-beta-actin showed very weak recovery into stress fibers (Fig 4A and 4B) and treatment with 7 μM jasplakinolide eliminated the minimal recovery of fluorescent actin into the FRAP zone (Fig 4A, inset). Although the recovery curve (Fig 4B) shows an ~50% reduction in the mobile fraction (Fig 4B, 4D, 4E and 4F) (S4 Table) this does not reflect true recovery into stress fiber bundles, but rather a recovery in fluorescence of the cytosolic G-actin pool (Fig 4A inset). Thus, treatment with jasplakinolide essentially eliminates the small amount of recovery of actin into stress fibers after photobleaching (Fig 4A).

We then investigated the kinetics of continuous diffusion as opposed to active binding of a tagged construct in our assay using Lifeact-RFP, which has extremely transient binding to actin. MEFs were transfected with Lifeact-RFP and FRAP analysis was performed on stress fiber regions in the presence and absence of 7 μM jasplakinolide. FRAP of Lifeact-RFP results in an almost instantaneous recovery in both control and drug treated conditions (Fig 4C). This is in agreement with the highly diffusive behaviour and transient binding of Lifeact constructs to its target site on actin [19]. Interestingly, a small reduction in the mobile fraction from control to drug treated cells was observed (Fig 4D). This perhaps suggests the existence of a sub-population of Lifeact bound to actin filaments in the interior of stress fibers that cannot as readily exchange as Lifeact at the periphery of stress fibers.

**Fig 5:**
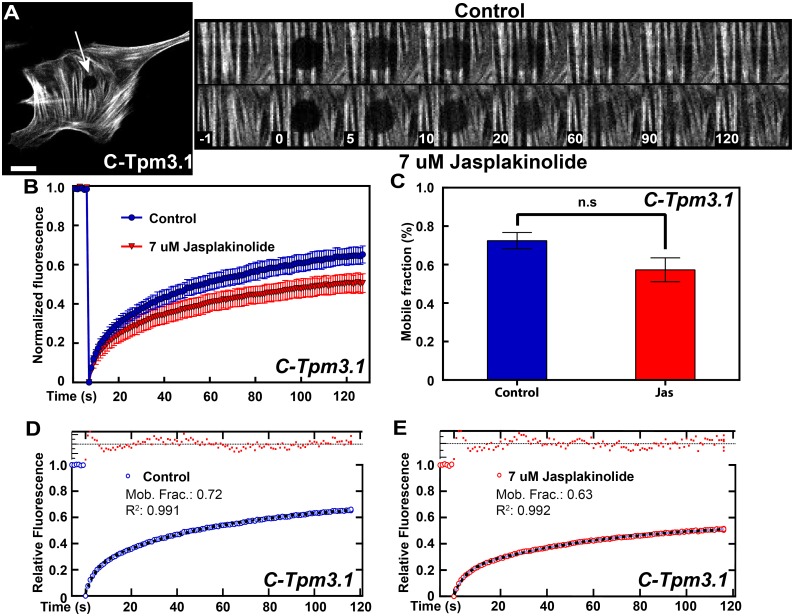
Tpm3.1 maintains constant and rapid cycling on stress fibers in the presence of jasplakinolide. (A) Representative image and FRAP sequence of MEFs transfected with C-Tpm3.1. FRAP zone indicated by white arrow. Top panel: FRAP sequence of untreated control cells. Bottom panel: FRAP sequence after treatment with 7 μM jasplakinolide. (B) FRAP curves of C-Tpm3.1 in control and drug-treated conditions. (C) Mobile fraction of control and drug-treated condition (see also S5 Table). (D,E) Curve fits for C-Tpm3.1 in control (D) and drug-treated condition (E). Data obtained from 3 separate experiments, 3–8 cells per experiment. Error bars are +/- *SEM*. Scale bars = 10 μm.

Having determined that treatment with 7 μM jasplakinolide significantly impacts actin turnover on stress fiber structures, we then sought to determine the recruitment kinetics of tagged Tpm3.1. MEF cells were transfected with constructs encoding C-Tpm3.1. Regions of stress fibers were photobleached prior to addition of jasplakinolide to obtain control curves (Fig 5A, inset). Jasplakinolide was then added to cells and the same cells were immediately photobleached at a different site (Fig 5A, inset). Intriguingly, jasplakinolide had a minimal effect on tagged Tpm3.1 recruitment into stress fibers (Fig 5A, inset), where only a small but not significant reduction in the mobile fraction was observed (Fig 5B, 5C, 5D and 5E) (S5 Table). As global actin turnover is significantly inhibited in the presence of the drug (Fig 4A and 4B), we conclude that Tpm3.1 is constantly undergoing dynamic exchange on actin filaments that is independent of actin filament dynamics. This is also apparent from inspection of the images of actin and Tpm3.1 recovery in Figs 4A vs 5A. Since Tpms bind actin through weak ionic interactions [36, 37] it may be logical that Tpm3.1 on filaments undergo changes independent of actin. The small reduction in mobile fractions of tagged Tpm3.1 (Fig 5B and 5C) and Lifeact-RFP (Fig 4C and 4D) in the jasplakinolide-treated conditions perhaps suggests that jasplakinolide is inhibiting the translocation and/or polymerization of a dynamic sub-population of actin filaments that operates in stress fibers, a hypothesis which is in agreement with what has been reported for cortical actin [8].

In conclusion, our results provide evidence that cytoskeletal Tpm3.1 binding to actin may be independent, at least in part, of actin dynamics. It is possible that the presence of the N- and C-terminal tags on Tpm3.1 impact the observed exchange kinetics in these experiments. It has been demonstrated previously that deletion of a single C-terminal amino acid can abolish Tpm binding to actin in yeast [38]. However, we have shown that expression of N-terminal tagged Tpm3.1 can supress an increase in cell speed induced in Tpm3.1 KO mouse embryo fibroblasts [31] indicating that the presence of an N-terminal tag does not completely abolish normal Tpm3.1 function. It is also possible that the replacement kinetics we observe primarily reflects the addition of tagged Tpm3.1 to the ends of actin filaments within filament bundles; however, the similar levels of accumulation of the tagged and endogenous Tpm3.1 make this unlikely (Fig 1C). Thus, it appears that Tpm3.1 dimers located within a filament are able to break two head-to-tail overlap interactions with adjacent dimers in the polymer together with their interaction with actin in order to exchange with ‘free’ Tpm3.1. While each of these interactions is of low affinity [1, 2], it seems likely that a source of energy may be required to weaken these interactions and promote the exchange reaction.

## Supporting Information

S1 Fig Spatial recovery of tagged Tpm3.1 is consistent with exchange rather than a diffusive transport process. (A,B) Simulated spatial recovery profile for (A) diffusion and (B) exchange reaction based recovery showing (top) kymographs of fluorescent intensity and (bottom) spatial profile of recovery at selected timepoints with cartoon illustrating relationship to bleached region. Black arrows indicate change in fluorescent intensity over time. Recovery due to diffusion shows an increase in the width of the bleached zone during the recovery due to motion of bleached and unbleached molecules from the surrounding areas while recovery due to an exchange reaction shows no change in the width of the bleached zone during the recovery. (C,D) Measured radially averaged spatial recovery profiles, for (C) N-Tpm3.1 and (D) C-Tpm3.1 averaged over n = 13 and n = 9 cells, respectively. (E,F) Fitted width of recovery profile over time for (E) N-Tpm3.1 and (F) C-Tpm3.1. Error bars indicate confidence interval on fit.
(TIF)

S1 Movie Movement of stress fibers within cells. MEFs transfected with EYFP-Tpm3.1 were imaged every 1 s for 5 min. The region of the cell highlighted with the red circle was bleached at 6 s. Movie is displayed at 50 fps.
(AVI)

S1 Table Half-times from double-exponential fits of N- and C-Tpm3.1 recovery in transfected MEFs. (DOCX)

S2 Table Half-times from double-exponential fits of N- and C-Tpm3.1 recovery in transfected rat acinar cells. (DOCX)

S3 Table Half-times from double-exponential fits of Lifeact-RFP recovery in control and drug-treated conditions. (DOCX)

S4 Table Half-times from double-exponential fits of GFP-actin recovery in control and drug-treated conditions. (DOCX)

S5 Table Half-times from double-exponential fits of C-Tpm3.1 recovery in control and drug-treated conditions. (DOCX)
