# Supplementary figures and images for: Recruitment Kinetics of Tropomyosin Tpm3.1 to Actin Filament Bundles in the Cytoskeleton Is Independent of Actin Filament Kinetics
Source: PLoS One. 2016 Dec 15;11(12):e0168203. doi: 10.1371/journal.pone.0168203 (PMC5158027; doi:10.1371/journal.pone.0168203)

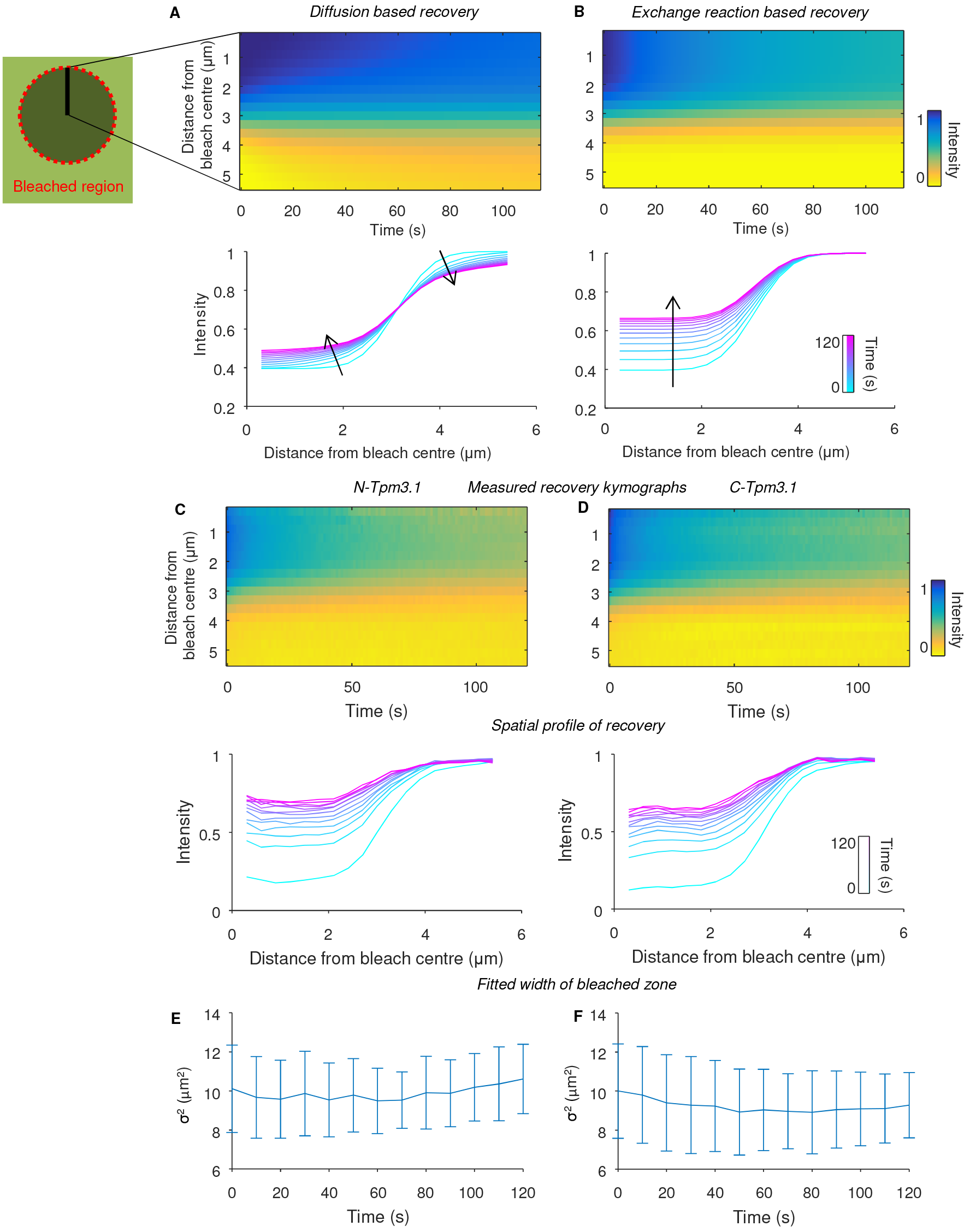

Supplement: S1 Fig — (A,B) Simulated spatial recovery profile for (A) diffusion and (B) exchange reaction based recovery showing (top) kymographs of fluorescent intensity and (bottom) spatial profile of recovery at selected timepoints with cartoon illustrating relationship to bleached region. Black arrows indicate change in fluorescent intensity over time. Recovery due to diffusion shows an increase in the width of the bleached zone during the recovery due to motion of bleached and unbleached molecules from the surrounding areas while recovery due to an exchange reaction shows no change in the width of the bleached zone during the recovery. (C,D) Measured radially averaged spatial recovery profiles, for (C) N-Tpm3.1 and (D) C-Tpm3.1 averaged over n = 13 and n = 9 cells, respectively. (E,F) Fitted width of recovery profile over time for (E) N-Tpm3.1 and (F) C-Tpm3.1. Error bars indicate confidence interval on fit. (TIF) [file pone.0168203.s001.tif]
