# Supplementary material for: Recruitment Kinetics of Tropomyosin Tpm3.1 to Actin Filament Bundles in the Cytoskeleton Is Independent of Actin Filament Kinetics
Source: PLoS One. 2016 Dec 15;11(12):e0168203. doi: 10.1371/journal.pone.0168203 (PMC5158027; doi:10.1371/journal.pone.0168203)
Supplement: S1 Table — (DOCX) [file pone.0168203.s003.docx]

**S1 Table. Half-times from double-exponential fits of N- and C-Tpm3.1 recovery in transfected MEFs.**

| **Half-times** | **N-Tpm3.1** | **Fractional contribution (%)** | **C-Tpm3.1** | **Fractional contribution (%)** |
| --- | --- | --- | --- | --- |
| **τ1** | 3.1 s (± 0.6) | 24 | 3.3 s (± 0.8) | 31 |
| **τ2** | 64.6 s (± 14.9) | 76 | 40.2 s (± 3.2) | 69 |

Data from *n* = 6 experiments
