# Supplementary material for: Recruitment Kinetics of Tropomyosin Tpm3.1 to Actin Filament Bundles in the Cytoskeleton Is Independent of Actin Filament Kinetics
Source: PLoS One. 2016 Dec 15;11(12):e0168203. doi: 10.1371/journal.pone.0168203 (PMC5158027; doi:10.1371/journal.pone.0168203)
Supplement: S2 Table — (DOCX) [file pone.0168203.s004.docx]

**S2 Table. Half-times from double-exponential fits of N- and C-Tpm3.1 recovery in transfected rat acinar cells.**

| **Half-times** | **N-Tpm3.1** | **Fractional contribution (%)** | **C-Tpm3.1** | **Fractional contribution (%)** |
| --- | --- | --- | --- | --- |
| **τ1** | 2.9 s (± 0.7) | 31 | 1.9 s (±0.9) | 26 |
| **τ2** | 45.3 s (± 6.8) | 69 | 20.5 s (± 5.9) | 74 |

Data from *n* = 3-4 animals
