# Supplementary material for: Recruitment Kinetics of Tropomyosin Tpm3.1 to Actin Filament Bundles in the Cytoskeleton Is Independent of Actin Filament Kinetics
Source: PLoS One. 2016 Dec 15;11(12):e0168203. doi: 10.1371/journal.pone.0168203 (PMC5158027; doi:10.1371/journal.pone.0168203)
Supplement: S3 Table — (DOCX) [file pone.0168203.s005.docx]

**S3 Table. Half-times from double-exponential fits of Lifeact-RFP recovery in control and drug-treated conditions.**

| **Half-times** | **Control** | **Fractional contribution (%)** | **Jasplakinolide** | **Fractional contribution (%)** |
| --- | --- | --- | --- | --- |
| **τ1** | 0.6 s (± 0.2) | 64 | 0.4 s (± 0.38) | 52 |
| **τ2** | 5.9 s (± 1.5) | 36 | 3.9 s (± 2.6) | 48 |
