# Supplementary material for: Recruitment Kinetics of Tropomyosin Tpm3.1 to Actin Filament Bundles in the Cytoskeleton Is Independent of Actin Filament Kinetics
Source: PLoS One. 2016 Dec 15;11(12):e0168203. doi: 10.1371/journal.pone.0168203 (PMC5158027; doi:10.1371/journal.pone.0168203)
Supplement: S4 Table — (DOCX) [file pone.0168203.s006.docx]

**S4 Table. Half-times from double-exponential fits of GFP-actin recovery in control and drug-treated conditions.**

| **Half-times** | **Control** | **Fractional contribution (%)** | **Jasplakinolide** | **Fractional contribution (%)** |
| --- | --- | --- | --- | --- |
| **τ1** | 2.4 s (± 0.4) | 36 | 1.4 s (± 0.9) | 33 |
| **τ2** | 65.5 s (± 12) | 64 | 35.7 s (± 22) | 67 |
