# Supplementary material for: Recruitment Kinetics of Tropomyosin Tpm3.1 to Actin Filament Bundles in the Cytoskeleton Is Independent of Actin Filament Kinetics
Source: PLoS One. 2016 Dec 15;11(12):e0168203. doi: 10.1371/journal.pone.0168203 (PMC5158027; doi:10.1371/journal.pone.0168203)
Supplement: S5 Table — (DOCX) [file pone.0168203.s007.docx]

**S5 Table. Half-times from double-exponential fits of C-Tpm3.1 recovery in control and drug-treated conditions.**

| **Half-times** | **Control** | **Fractional contribution (%)** | **Jasplakinolide** | **Fractional contribution (%)** |
| --- | --- | --- | --- | --- |
| **τ1** | 3.0 s (±0.4) | 28 | 3.1 s (± 0.4) | 30 |
| **τ2** | 42.3 s (± 7) | 72 | 43.9 s (± 13) | 70 |

Data from *n* = 3 experiments
